# Recovering the susceptibility of antibiotic-resistant bacteria using photooxidative damage

**DOI:** 10.1073/pnas.2311667120

**Published:** 2023-09-20

**Authors:** Jennifer M. Soares, Vladislav V. Yakovlev, Kate C. Blanco, Vanderlei S. Bagnato

**Affiliations:** ^a^Institute of Physics of São Carlos, University of São Paulo, São Carlos 13566-590, Brazil; ^b^Biomedical Engineering, Texas A&M University, College Station, TX 77840

**Keywords:** photodynamic inactivation, antimicrobial resistance, *Staphylococcus aureus*, antibiotic failures

## Abstract

Multidrug-resistant bacteria are one of the most serious threats to infection control. Few new antibiotics have been developed; however, the lack of an effective new mechanism of their action has worsened the situation. Photodynamic inactivation (PDI) can break antimicrobial resistance, since it potentiates the effect of antibiotics, and induces oxidative stress in microorganisms through the interaction of light with a photosensitizer. This paper addresses the application of PDI for increasing bacterial susceptibility to antibiotics and helping in bacterial persistence and virulence. The effect of photodynamic action on resistant bacteria collected from patients and bacteria cells with induced resistance in the laboratory was investigated. *Staphylococcus aureus* resistance breakdown levels for each antibiotic (amoxicillin, erythromycin, and gentamicin) from the photodynamic effect (10 µM curcumin, 10 J/cm^2^) and its maintenance in descendant microorganisms were demonstrated within five cycles after PDI application. PDI showed an innovative feature for modifying the degree of bacterial sensitivity to antibiotics according to dosages, thus reducing resistance and persistence of microorganisms from standard and clinical strains. We hypothesize a reduction in the degree of antimicrobial resistance through photooxidative action combats antibiotic failures.

## Significance

Bacteria resistant to antibiotics are now one of the great challenges for the control of infections. Many unsuccessful attempts have been made toward breaking such resistance through chemical modifications or addition of other conjugated compounds. This paper addresses the ability of photo-oxidation to reduce bacterial resistance, making them again sensitive to antibiotics, and explores relevant facts that may allow for the return of bacterial susceptibility to resistant organisms.

Recurrent infections after therapy were reported 12 y after the discovery of antibiotics, indicating the existence of multidrug-resistant bacteria (MRB). Microbial resistance to antibiotics is estimated as 225 million cases by 2030 with up to 10 million annual deaths by 2050 (1–4). Aggravating factors include a few new classes of antimicrobials and a few improvements in the molecules under development (5, 6). MRB has intrinsic mechanisms, normally acquired by mutations in adaptive conditions, which prevent the action of drugs by not absorbing, or degrading, and even expelling them from the cell. The main mechanisms for antibiotic inactivation are reduced microbial membrane permeability, increased efflux pump activity, changes in antibiotic binding targets, and synthesis of microbial enzymes that react with antimicrobials. Those structural and metabolic alterations increase minimum inhibitory concentrations (MIC), which may reach unfeasible values for a safe and effective treatment against microorganisms (7, 8). Alterations that occur in infections are often associated with failures in the response to antibiotic therapies.

**Fig. 1.**
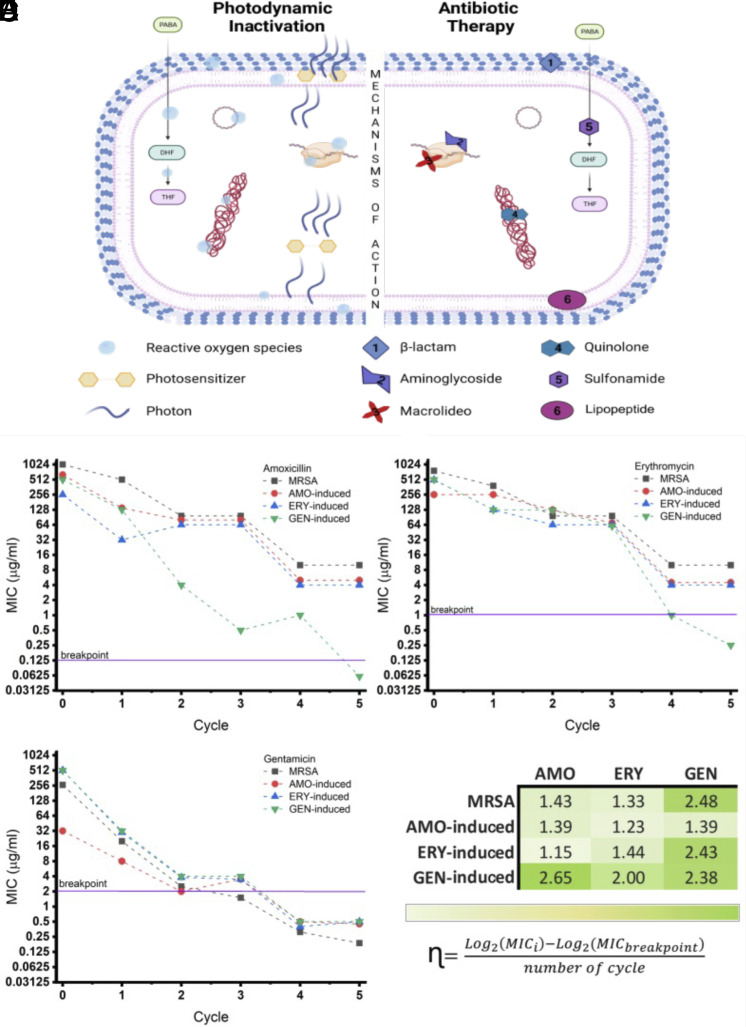
Combination of PDI with antibiotics. PDI cycle of 10 µM curcumin, 10 J/cm^2^ at 450 nm with antibiotics. The strains evaluated were collected from patients (MRSA) and induced resistance of *S. aureus* (ATCC25923) by culturing ¼ MIC of AMO (0.063 μg/mL), ERY (4 μg/mL), and GEN (0.125 μg/mL). (*A*) PDI occurs and acts through the absorption of light by the PS internalized by the bacteria, which produces reactive oxygen species (bubbles in the picture) that oxidize several molecular and cellular structures, triggering bacterial inactivation. On the other hand, antibiotic action can be either bactericidal or bacteriostatic and is dependent on the structure of the antibiotic molecule that specifically interacts with cellular components. Increased susceptibility to antibiotics with the determination of MIC of each strain referring to (*B*) amoxicillin, (*C*) erythromycin, and (*D*) gentamicin. (*E*) Values of ŋ are indicative of a rate of resistance breakdown by the action of partial PDI for each strain and antibiotic evaluated. Breakpoint defined by BrCAST/EUCAST (13). Data are the median of experimental triplicates. Plots using *y*-axis log_2_ scale.

The main strategy for the fight against MRB infections has been the development of new antimicrobial drugs. However, regardless of their action, failures in antibiotic treatments tend to occur (9). Proof of principles for breaking down antibiotic resistance in methicillin-resistant *Staphylococcus aureus* has been conducted through the combination of antimicrobials with photodynamic treatments, which consistently decreases *S. aureus* resistance and improves susceptibility to antibiotic treatment (10). Such a development is an alternative for overcoming part of the growing challenge of making MRB susceptible to regular antibiotics. Curcumin, a photosensitizer (PS) derived from the extract of *Curcuma longa* root, with photodynamic action, has shown to potentiate some antibiotics by affecting the bacterial membrane, due to its lipophilic nature, and other cellular components (11, 12). Antibiotics act on a certain bacterial cell compartment, depending on their type, whereas PDI acts in the entire bacterial cell, causing multiple damages, as schematically shown in Fig. 1*A*. Apart from the synergistic effects obtained with the combined use of antibiotics and PDI (11), the dosage of antimicrobials must be reduced toward increasing the application of antibiotics and mitigating their failures. This study aims at demonstrating such facts.

An investigation on the level of breakdown of antimicrobial resistance to antibiotics with the use of PDI and the minimum time necessary for killing the population is proposed. A PDI triggers oxidative stress in microorganisms due to the light absorption by the photosensitizing molecule (14) and the reactive oxygen species formed by PDI, such as superoxide radical, hydrogen peroxide, hydroxyl radical, and singlet oxygen, interact with all biomolecules so that no molecular strategy attenuates the photodynamic action and selects bacteria, which is a notable differential concerning antibiotic therapy (15). A unique characteristic of the recent study is the demonstration of whether a bacterium has become resistant to a specific antibiotic, according to the following questions: What are the resistance factors? Once resistance has been broken, how do other antibiotics work? Once susceptible again to antimicrobials, when does the resurgence of resistance occur during the reduction in an infection?

## Results

### Recovery Susceptibility to Antibiotics.

Fig. 1 displays the effect of partial PDI cycles on the MIC of resistant *S. aureus* strains every 6 h from both patient samples (MRSA) and induced *S. aureus* resistance (ATCC25923) by culturing with ¼ MIC of amoxicillin (AMO, 0.063 μg /mL), erythromycin (ERY, 4 μg/mL), and gentamicin (GEN, 0.125 μg/mL). Cycle zero corresponds to the initial MIC value of the strains from the frozen stock inoculum. MRSA showed a high MIC value for the three antibiotics, with a 1,024 μg/mL concentration as the maximum value to be detected. Regardless of the antibiotic used to induce resistance, the MIC values of the offspring strains were above the breakpoint for AMO, ERY, and GEN susceptibility (13). Partial PDI with 10 μM curcumin and 10 J/cm^2^ reduces 2.81 log (CFU/mL), representing the effect of partial PDT observed throughout this study (11)

The resistance of *S. aureus* strains induced by GEN after five cycles of partial PDI showed MIC below the breakpoint, classifying them as sensitive to AMO, ERY, and GEN. Fig. 1*B*, after five cycles of partial PDI, shows decreases in AMO MIC of 102; 128; 64, and 8,533-fold for MRSA, *S. aureus* AMO-induced, ERY-induced, and GEN-induced, respectively, were observed. Fig. 1*C* indicates the MIC values decreased to 77; 64; 128, and 2,048-fold for MRSA and *S. aureus* AMO-induced, ERY-induced, and GEN-induced, respectively. The application of partial PDI was efficient in the action of GEN, remaining below the breakpoint for all strains after two cycles of MIC reduction (Fig. 1*D*). After five partial PDI cycles, the new GEN MIC was 0.1875 μg/mL for MRSA and 0.5 μg/mL for AMO-induced, ERY-induced, and GEN-induced *S. aureus*.

Fig. 1*E* displays the values from the analysis of the resistance breakdown rate of the photooxidative action of partial PDI in cycles, defined as ŋ. The ŋ indicates the efficiency of the resistance break based on the experimental data, i.e., the rate at which the initial MIC tends to reach the breakpoint to be classified as sensitive. The parameter indicates GEN-induced *S. aureus* strain responds more effectively to the action of partial PDI cycles in increasing susceptibility to AMO > GEN > ERY. Since GEN showed the highest ŋ values, its recovery of susceptibility was more effective for MRSA > GEN-induced > ERY-induced > AMO-induced. A high ŋ represents higher efficiency in the viability of antibiotic recovery, i.e., the action of PDT enables the use of antibiotics in strains where their use would be less effective.

### MIC of the Descending Cell After PDI Cycles.

**Fig. 2.**
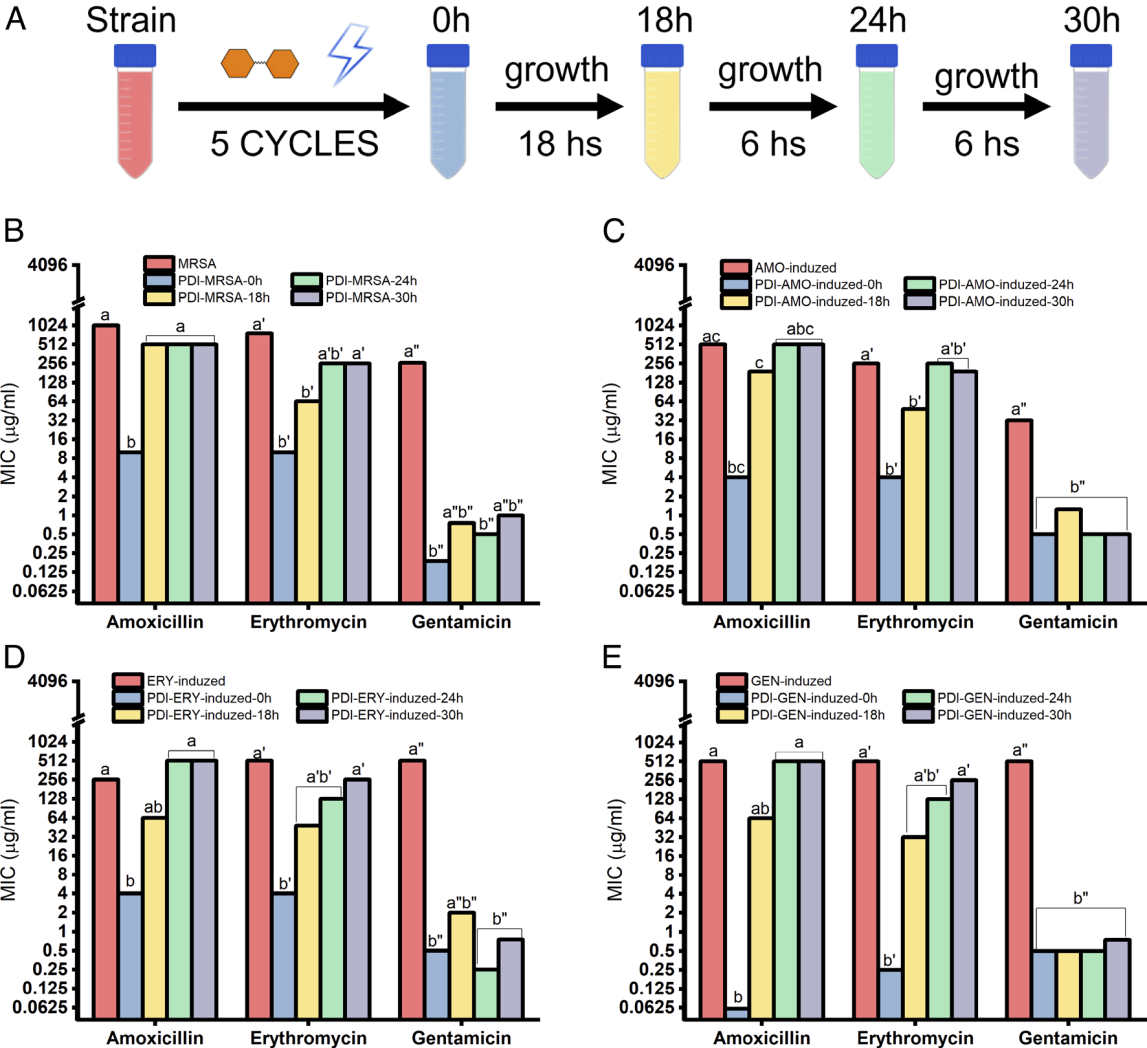
MIC of *resistant S. aureus* strains and descendants. (*A*) Scheme of the methodology for the determination of the MIC of the initial strain at 18, 24, and 30 h after five cycles of partial PDI. (*B*) Clinically isolated MRSA, (*C*) Amo-induced, (*D*) ERY-induced, and (*E*) GEN-induced. Data are the median of experimental triplicates. Different letters indicate statistical difference for *P* < 0.05, and equal letters denote no statistical difference. The analysis considered each antibiotic individually. Plots using *y*-axis log_2_ scale.

Fig. 2 shows the increased bacterial susceptibility obtained with a partial PDI treatment after five cycles was maintained in progeny cells cultured directly from the colony in BHI medium with no treatment. According to Fig. 1, after five cycles of partial PDI, MIC decreased for clinical and induced resistant strains. The red and blue bars in Fig. 2 denote bacteria before and after five partial PDI cycles, respectively. For all strains evaluated, the MIC obtained in the 18 h culture was lower than the MIC_0_ (red bar) of MRSA and *S. aureus* AMO-induced, ERY-induced, and GEN-induced strains for AMO, ERY, and GEN antibiotics. Transferring the culture to a fresh and cultivated medium and maintaining it there until 24 h and 30 h caused AMO and ERY to return to the MIC_0_ of resistant strains, i.e., the one before the treatment of partial PDI cycles. However, gentamicin kept the MICs at lower values compared to the MIC_0_ of resistant strains.

After 30 h cultivation, the MIC of PDI-MRSA strain (Fig. 2*B*) was 2, 3, and 264 times lower than MIC_0_ for AMO, ERY, and GEN, respectively. According to Fig. 2*C*, the MIC of PDI-AMO-induced strain was 1.3- and 64-fold lower for ERY and GEN, whereas that of AMO recovered the baseline value of the resistant strain. The MIC of AMO for PDI-ERY-induced (Fig. 2*D*) was twice MIC_0_, but 2 and 683 times lower for ERY and GEN, respectively. Regarding PDI-GEN-induced (Fig. 2*E*), after 30 h cultivation, the MIC of the bacterial population was 2 and 683 times lower than MIC_0_ of ERY and GEN, respectively, but recovered the MIC_0_ value for AMO.

### Effect of PDI on Persistence.

**Fig. 3.**
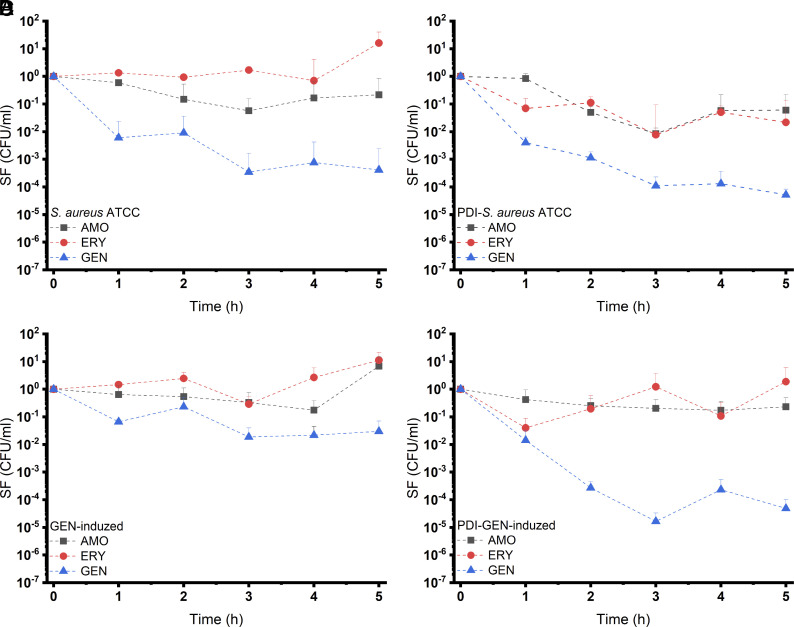
Bacterial SF with the cultivation of 10 MIC_0_ of AMO, ERY, and GEN of strains. (*A*) *S. aureus* ATCC 25923, (*B*) previous application of partial PDI (10 μM curcumin, 10 J/cm^2^ at 450 nm) for the cultivation of *S. aureus* ATCC 25923, (*C*) *S. aureus* induced by GEN, and (*D*) previous application of partial PDI (10 μM curcumin, 10 J/cm^2^ at 450 nm) for the cultivation of *S aureus* induced by GEN.

The minimum duration for killing 99% of the population (MDK_99_) is a complementary parameter that indicates sensitive, persistent, and resistant bacteria. Moderate MDK_99_ values characterize a persistent strain, i.e., although they are sensitive bacteria, the time for the antibiotic to exert an effect is longer. Fig. 3 displays the survival fraction (SF) of *S. aureus* (ATCC 25923) and GEN-induced strains with and without the application of partial PDI. According to Fig. 3*A*, MDK_99_ could be obtained in a 5 h experimental data period for AMO and ERY, although the strain is sensitive to AMO. However, for GEN, the value was 2.4 h. The previous application of partial PDI in *S. aureus* (Fig. 3*B*) decreased MDK_99_ for GEN and ERY, being 1.9 h and 4.8 h, respectively, while for AMO, MDK_99_ is mathematically 5.5 h. The SF decrease rate was faster for the previous application of partial PDI from 0.3 to 0.8 times. The behavior of GEN-induced *S. aureus strain*, which is resistant to all antibiotics, displayed over time was that of a surviving fraction whose MDK_99_ is longer than 5 h. However, the previous application of partial PDI in strains induced by GEN (Fig. 3*D*) showed the behavior of the SF decreased 0.1- to 1.3-fold for all antibiotics evaluated, enabling an experimental determination of MDK_99_ for GEN in 1.8 h.

## Discussion

This study aims to demonstrate that photodynamic action is essential in cases of antibiotic failure and an excellent adjuvant to antimicrobial therapy. An increase or a full recovery of susceptibility to antibiotics (Fig. 1) can be a way to prolong the useful life of recent classes of antibiotics, which is essential for avoiding infection control collapses. The chance of new antibiotics being approved by the FDA for clinical phase III studies is 6 out of 10, and the probability of the ones approved being a new antibiotic class is only 25% (6). Such an estimate implies a low probability of solving the bacterial resistance problem since most new antimicrobials tend to derive from existing classes. A strategy usually adopted clinically is the association of different antimicrobial classes for reducing treatment doses and synergistic responses. However, in some cases, both antagonistic response and exposure to more than one class of antibiotics limit treatment options, reducing the time of use of that class (16–18). The combination of two or more antimicrobials can induce drug tolerance of bacterial strains, i.e., prolonged limited-time survival of bacteria under lethal conditions (19), thus hampering the eradication of infection due to reinfections and the fact approximately 80% are biofilm infections (20, 21). The search for techniques that combat antimicrobial resistance mechanisms is therefore essential.

Bacterial resistance to antimicrobials can develop from artificial selection processes, according to which inadequate exposures of drugs to microorganisms eliminate susceptible cells and select resistant bacterial ones (22), as shown in *SI Appendix*, Fig. S1. Nevertheless, induction by a certain class of antibiotics also causes resistance to other classes, as indicated by our results. A strategy to enhance antimicrobial effects is to associate techniques such as PDI, which significantly decreases the MIC values independently of the original resistance.

Our previous studies demonstrated the PDI action potentiates the antibiotic effect with the use of methylene blue PS (10) and curcumin (11) with different antibiotics, corroborating others according to which the order of application among therapies, PS choice, and class of antibiotics can determine synergistic responses (12, 17, 18, 23–30)

The results in Fig. 1 show the susceptibility increase for three classes of antibiotics is effective in the resistant bacterial population when submitted by PDI, whether clinically isolated or induced in laboratory. Although the procedure was not optimized, optimization is believed to enhance results. Modifications in both interval and PDI duration can break resistance through photodynamic action in all groups, which was achieved only for GEN (Fig. 1*D*) and for the strain of *S. aureus* GEN*-*induced in the other antibiotics, obtaining MIC below the breakpoint. Gentamicin is well known to pass through the bacterial membrane and bind to rRNA in an oxygen-dependent active transport, preventing the formation of proteins (31). If the threshold is not reached (Fig. 2), the predominant surviving population remains as cells not susceptible to antimicrobials, so that in the next generations of the population, the final MIC is greater than or equal to MIC_0_, i.e., with no application of photodynamic action. When the breakpoint is exceeded, the probability of a cell’s predominance being susceptible to antimicrobials in the population increases; consequently, the MIC of the next generations may not increase significantly, as in the case of the use of GEN (Fig. 2).

GEN stands out regarding breakage of resistance and maintenance of susceptibility after recovery for the different strains studied. As an aminoglycoside, it acts on the 30S ribosome subunit, whereas ERY, a macrolide, acts on the 50S ribosomal subunit (31, 32). A combination of PDI, GEN, and ERY implies a higher probability of bacteria remaining, with damage to the ribosome (an essential organelle for maintaining cell activities). Such damage is slower to recover than damage to the cell wall, which is the most cumulative damage from the use of AMO, β-lactam (33). Since the interval between PDI applications was 6 h and significantly longer than the time for new generations to be produced, the data revealed a permanent effect of decreasing MIC when photooxidative plus antibiotic damage is more difficult to be repaired by the cell’s regulatory mechanism. Furthermore, a higher ŋ implies the damage rate exceeds regeneration and the breakdown of resistance tends to be more permanent. The opposite occurs when ŋ is low.

The distinct response to the combination of PDI and antibiotic between strains of *S. aureus* and the different classes of antibiotics is not universal for each trio of bacteria, PS, and antibiotic. Moreover, our results were influenced by the growth phase of the bacteria (Fig. 2), after increasing susceptibility to antimicrobials and transferring those strains to culture with no additional treatment. When the bacterium reaches the stationary phase (18 h), it shows more susceptibility than when it is in the logarithmic phase (6 h recultivation until 24 h and 30 h).

On the other hand, PDI not only increases susceptibility to antibiotics but also decreases MDK_99_ (Fig. 3), which is essential for the action of those drugs, since they act mainly during the growth period. Persistent cells tend to show a higher rate of smaller growth (34); therefore, the MDK_99_ reduction promoted by PDI combats another aspect of antimicrobial failure. In persistence, microbial populations do not increase in the presence of the antimicrobial, and population growth is resumed only after drug withdrawal. In the persistence experiment conducted, the microbial pathogenesis of resistance-induced and susceptible bacteria was evaluated due to the need to prolong the treatment, considering the possibility of relapse after therapy. In such recurrent cases in patients, alternatives are used toward the effectiveness of the treatment, which can also increase side effects. Some phenotypic characteristics in a subpopulation, including persistent bacteria that survive in high antibiotic concentrations reinfecting patients, can affect antimicrobial therapy, hence, antibiotics’ ineffectiveness (35, 36). Furthermore, the severe symptoms of infection are due to virulence factors such as toxins and biofilm formation, whose expression can be stimulated by the dosage of the antibiotic (37, 38). Our study also indicates improvements in those aspects.

A previous study demonstrated that treatments, whether antibiotic monotherapies, PDI, or combined ones, alter the bacterial phenotype (11). Through adhesins, bacteria bind to a substrate as tissue cells, causing the development of an infection and bacterial targeting to a specific substrate, which is the first step toward the formation of a complex three-dimensional structure, i.e., a biofilm, characteristic of persistent, chronic, and recurrent bacterial infections (39). According to *SI Appendix*, Fig. S2, biofilm shows a higher potential for formation when strains are more resistant (higher MIC) to antimicrobial-sensitive cells. However, after the fifth cycle of PDI, descendant strains decrease such virulence expression, once again corroborating the photooxidative action on the cells, which modifies both structures and cellular metabolism when not eliminated. PDI, which aims to break down resistance, can show positive indices after five cycles in collaborating with the treatment of bacterial biofilm infections that resist host immune responses and antibiotics. Rudrappa and Bais (40) observed that curcumin has an anti-infective action by inhibiting *Pseudomonas aeruginosa* biofilm. The reduction in biofilm formation is relevant for the treatment of bacterial infections since, in addition to patient deterioration, biofilms tend to increase up to 1,000 times antimicrobial resistance. Some studies have indicated that biofilms are not necessarily resistant (41) to antibiotics and the environment favors the persistence of bacterial cells to antimicrobials (42).

Photodynamic action not only is a possible technique to improve infection control but also modifies the bacterial susceptibility degree to antimicrobials by decreasing both resistance to below breakpoints, MDK_99_ and controls the biofilm formation, which is an important bacterial virulence factor. In those three main points related to antibiotic failure, PDI associated with conventional treatments proved an adequate and beneficial treatment for future applications in bacterial infections and a response to the demand of the World Health Organization for alternatives. However, several other points on the long-term response of such a combined treatment must be elucidated.

Finally, the main mechanisms for photodynamic to reduce bacterial resistance to antibiotic remain to be determined. Nevertheless, the observations made enable the establishment of a hypothesis. When bacteria acquire resistance to a certain antibiotic, modifications occur both in its metabolisms and in permeability through the membrane. Enzymatic reactions may destroy the antibiotic molecule or the efflux pump may expel such molecules. The oxidation introduced by photodynamic action can interfere with those mechanisms decreasing their activity, thus breaking down barriers to antibiotic action.

According to findings such as reduced antimicrobial resistance and increased antimicrobial susceptibility with PDI applied at regular intervals, PDI has proven an option for damaging or destroying microbial integrity. Our results have confirmed our hypothesis, providing an approach to address antibiotic failures using PDI and indicating the possibility of conjugating antibiotics with PDI such that the existent generations of antibiotics may turn back to being viable in the fight against infections. With time, the reversal resistance to the antibiotic is expected to be permanent so that the antibiotic alone can again be a good solution. Further research is necessary for a broader assessment of other antibiotics, microorganisms, and PSs for a full understanding of the mechanisms of action and interaction between PDT and antibiotics. Nevertheless, the combination of photodynamic oxidation with antibiotics may be a new modality of fighting infections in many situations, helping revert a difficult scenario of antimicrobial resistance.

## Materials and Methods

### Microorganism.

*S. aureus* strain (ATCC 25923) and methicillin-resistant *S. aureus* (MRSA) strain clinically isolated from patients (brasilian partner laboratory) were used. Bacteria were cultured aerobically in a Brain Heart Infusion (BHI) liquid medium overnight at 37 °C and 150 rpm. The inoculum was centrifuged at 3,000 rpm, and the pellet was suspended in phosphate-buffered saline (PBS) in the same initial volume and centrifuged again. The inoculum was standardized at 10^7^ to 10^8^ colony-forming units per milliliter (CFU/mL) by optical density at 600 nm. Regarding the experiments (*Minimum Inhibitory Concentration* and *Biofilm Biomass Quantification*), the inoculum was prepared by directly suspending bacterial colonies in PBS.

### Minimum Inhibitory Concentration.

Different concentrations of amoxicillin, erythromycin, and gentamicin sulfate were distributed in a 96-well plate in a Muller Hilton (MH) liquid medium. The bacterial inoculum was added to a 10^6^ CFU/mL final concentration in each well. After 24 h-incubation at 37 °C, resazurin was added and maintained for 4 h for the determination of the MIC of each antibiotic. For the determination of the MIC of descendant cells after PDI, the inoculum was cultivated in BHI for 18 h and then transferred to a fresh medium, completing 24 and 30 h of growth. Standardization in 10^6^ CFU/mL was followed for each interval of 18, 24, and 30 h.

### Partial PDI Cycle.

Standardized inoculum at 10^8^ CFU/mL of resistance-induced strains with ¼ MIC (*SI Appendix*, Development of antibiotic resistance) and MRSA was subjected to a partial PDI protocol, i.e., a partial inactivation of the bacterial population, which damaged less than 3 log(CFU/mL). The curcumin PS stock solution (PDT PHARMA®) was prepared in 5 mM ethyl alcohol, and the solution to be used was diluted in water for a 10 µM final contraction. 250 µL of bacteria + 250 µL of curcumin were added to a 24-well plate for each well and kept in the dark at 37 °C for 15 min. Subsequently, the plate was subjected to a 10 J/cm^2^ irradiation (4.10 min) by an LED-based device (Biotable ®) at 450 nm wavelength and 40 mW. The samples underwent two stages, namely, i) determination of MIC, described above, and ii) cultivation in BHI medium for 6 h intervals for the repetition of the protocol for five cycles. In the last step, the strains were frozen in a cryotube containing BHI medium and 20% glycerol. A new inoculum was then formed for the MIC determination.

### Persistence Test.

After standardization of the inoculum, the bacteria were transferred to 25 mL Falcon tubes containing MH medium with 10×MIC of amoxicillin, erythromycin, or gentamicin sulfate. A group previously subjected to the partial PDI protocol was incubated with 10 µM of curcumin and irradiated at 10 J/cm^2^, 450 nm on Biotable®. The surviving cells were then transferred to Falcon tubes with 25 mL of MH medium containing 10×MIC of the three antibiotics. Samples were collected, diluted, and seeded every hour for the CFU/mL determination.

### Statistical Analysis.

Experiments were conducted in biological triplicate (*N* = 9), and the Shapiro–Wilk test assessed data normality. Data described by normal distribution were plotted as mean with SD and analyzed by ANOVA, one way, and Tukey. Kruskal–Wallis and median tests were applied for nonparametric data, and the results were plotted as a median. *P* value < 0.05 was considered significant for all analyses. TIBCO Software Inc version 14 was used for the statistical analyses, and Origin Version 2022b plotted the graphics.

## Supplementary Material

Appendix 01 (PDF)

## Data Availability

All study data are included in the article and/or *SI Appendix*. For more information contact the authors.
