# Supplementary material for: Recovering the susceptibility of antibiotic-resistant bacteria using photooxidative damage
Source: Proc Natl Acad Sci U S A. 2023 Sep 20;120(39):e2311667120. doi: 10.1073/pnas.2311667120 (PMC10523486; doi:10.1073/pnas.2311667120)
Supplement: Supplementary file 1 — Appendix 01 (PDF) [file pnas.2311667120.sapp.pdf]

PNAS

Supporting Information for

Recovering the susceptibility of antibiotic-resistant bacteria using photooxidative damage

Jennifer M. Soares [1, 2], Vladislav V. Yakovlev[2], Kate C. Blanco[1,] and Vanderlei S. Bagnato[1, 2*]

[1] Institute of Physics of São Carlos, University of São Paulo – São Carlos - SP, Brazil

[2] Biomedical Engineering, Texas A&M University - College Station - TX, USA

Correspondent author: Vanderlei S. Bagnato

Email: vander@ifsc.usp.br

This PDF file includes:

Supporting text

Methods

Figures S1 to S2

Supporting Information Text

Result

Increased bacterial resistance to antibiotics

A continuous exposure to AMO (MIC_0 = 0.25 µg / mL), ERY (MIC_0 = 16 µg/mL), and GEN (MIC_0 = 0.5 µg/mL) at their corresponding concentrations of initial MIC (MIC_0), ½ MIC_0, and ¼ MIC_0 was administered to S. aureus (ATCC 25923) cultures, which were subsequently washed and suspended in a fresh medium containing identical subinhibitory antibiotic concentrations at 24-hour intervals. The MIC values remained constant in the first 72 h of cultivation (Figure S1.A) and, with AMO, increased between 160 and 384 times. Figure S1.B shows a decrease in MIC for ERY in the first 24 hours; however, in the remaining hours, MIC_0 linearly increased 2 to 4 times. The larger increase is due to culture concentrations of ½ MIC_0 and ¼ MIC_0 for AMO and ERY, whereas, for GEN (Figure S1.C), the result was independent of the concentration, with an increase of 64 times in MIC_0. After 72 h of culture, the MIC values for the three antibiotics significantly increased, proving the descendant strain resists AMO, ERY, and GEN, which represent the antimicrobial-resistant samples used in the subsequent stage.

Biofilm biomass quantification

Biofilm formation is one of the expressions of bacterial virulence. Biomass adhered to a substrate is measured by the amount of extracellular matrix by crystal violet absorption (570 nm) and the presence of bacterial cells by optical density (600 nm). Comparatively, in Figure S2, the strains whose resistance was induced by cultivation in ¼ MIC_0 of AMO, ERY, and GEN (Figure S1) showed, respectively, 20.4%, 2%, and 2.2% increases in biofilm biomass over the ancestral strain (ATCC). The biofilm biomass of the MRSA patient strain was also 15.3% greater than that of the ATCC strain. A previous application of partial PDI to resistant bacteria decreased the biofilm biomass adhered to the plate surface by 12.6%, 1.,8%, and 0.3% for AMO-induced, ERY-induced, ERY-induced, and MRSA compared to a non-previous application. The result in the figure illustrates potential differences in the expression of virulence factors between resistant and sensitive bacteria. Furthermore, the impact of PDI cycles is not limited to modifying susceptibility profiles; it also suggests potential antivirulence effects.

Methods

SI Development of antibiotic resistance

Initially, the inoculum standardized at 10^8 CFU/ml was transferred to a Falcon tube containing 6 ml of MH medium with antibiotic concentration varying from MIC, ½ MIC, and ¼ MIC. Cultivation was maintained aerobically for 24 h at 37°C and 150 rpm. Subsequently, the inoculum was centrifuged at 300 rpm and resuspended in MH for 10^8 CFU/ml standardization. The new inoculum was divided into two steps, namely i) determination of MIC (section **Minimum inhibitory concentration**) and ii) cultivation in 6 ml of MH medium with antibiotic concentration varying from MIC, ½ MIC, and ¼ MIC. The procedure was repeated for up to 3 cycles. Strains that showed MICs far above the breakpoint(15) were frozen in a cryotube containing BHI medium and 20% glycerol in the last step.

SI Biofilm biomass quantification

The inoculum was adjusted to 10^8 CFU/ mL and 200 µL were distributed in a 96-well plate incubated for 48 h at 37 °C. Subsequently, each well was washed twice with 200 µl of PBS. 100 µL of a 0.1% Crystal Violet dye solution in contact with the biofilm for 15 minutes were removed and each well was washed four times with 200 µL of distilled water. The plates were left at room temperature for 15 min for complete evaporation of water residues. Absolute ethyl alcohol (200 µL) was then added to each well for solubilizing the dye adhered to the matrix. The total biomass was quantified through the reading of optical density at 570 to 600 nm wavelength range. The higher absorbance results indicated a larger biomass production in the biofilm.

Figures

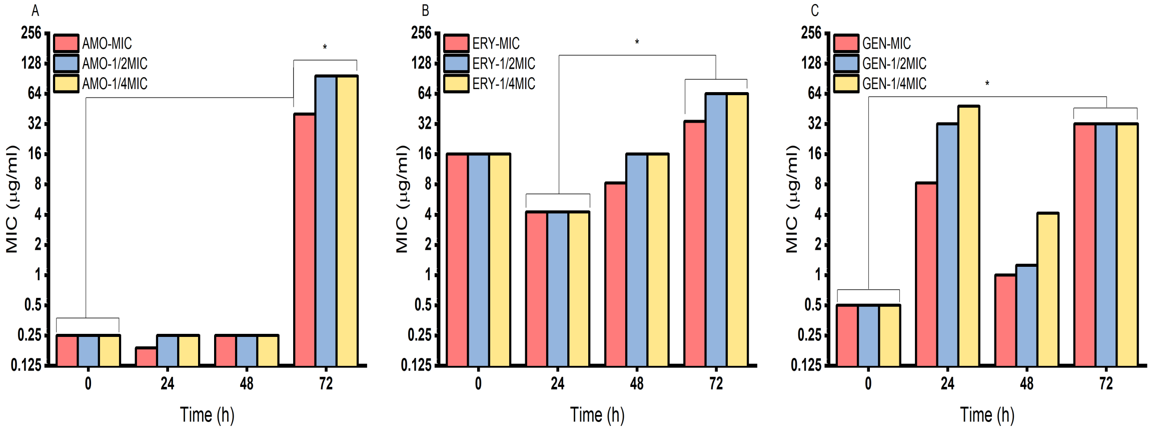

Figure S1. Minimum inhibitory concentration (MIC) after induction of antibiotic resistance with a culture of *S. aureus* (ATCC 25923) containing MIC_0, 1/2 MIC_0, and 1/4 MIC_0 for (**A**) amoxicillin, whose MIC_0 is 0.25 µg/mL, (**B**) erythromycin, whose MIC_0 is 16 µg/mL, and (**C**) gentamicin, whose MIC_0 is 0.5 µg/mL. Data are the mean of experimental triplicates. * Indicates significant statistical difference among the groups for $p<0.05$. Plots using y-axis Log_2 scale.

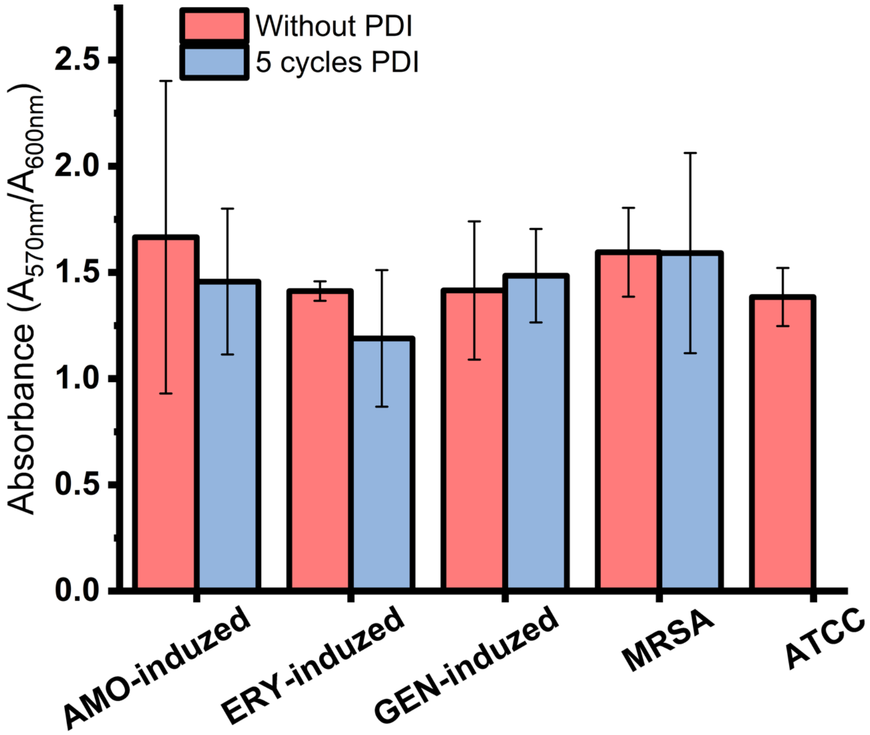

Figure S2. Quantification of adhered biofilm biomass. Normalized crystal violet absorbance (570 nm) by bacterial cell optical density (600 nm) for different bacterial strains of *S. aureus* (amoxicillin-induced, erythromycin-induced, gentamicin-induced, MRSA, and ATCC 25923). Strains with/without application of 5 cycles of partial PDI (10 μM of curcumin, 10 J/cm² at 450 nm) before biofilm induction.
